# ﻿Introduction of two novel species of *Hymenopellis* (Agaricales, Physalacriaceae) from Thailand

**DOI:** 10.3897/mycokeys.98.104517

**Published:** 2023-07-24

**Authors:** Allen Grace T. Niego, Naritsada Thongklang, Kevin D. Hyde, Olivier Raspé

**Affiliations:** 1 School of Science, Mae Fah Luang University, Chiang Rai 57100, Thailand; 2 Center of Excellence in Fungal Research, Mae Fah Luang University, Chiang Rai 57100, Thailand; 3 Iloilo Science and Technology University, La Paz, Iloilo 5000, Philippines; 4 Department of Biology, Faculty of Science, Chiang Mai University, Chiang Mai 50200, Thailand; 5 Key Laboratory for Plant Diversity and Biogeography of East Asia, Kunming Institute of Botany, Chinese Academy of Sciences, Kunming 650201, Yunnan, China; 6 Institute for Plant Health, Zhongkai University of Agriculture and Engineering, Haizhu District, Guangzhou 510225, China

**Keywords:** 2 new species, morphology, macrofungi, phylogeny, Southeast Asia, taxonomy

## Abstract

*Hymenopellis* is the most diverse genus in the group of oudemansielloid/xeruloid taxa (Physalacriaceae). This genus has a worldwide distribution with records mostly from Europe and America. Asian taxa are least represented. In this paper on *Hymenopellis* from Thailand, two novel species are introduced, and a *Hymenopellis* collection affine to *H.orientalis* is described. Macro and micromorphological characters are described. Maximum likelihood and Bayesian phylogenetic analyses were performed on combined ITS and nrLSU regions to confirm taxonomical placement and infer the phylogenetic affinities of the studied species. *Hymenopellisstraminea* sp. nov. is straw-yellow, with medium-sized basidiomata, abundant and diverse in form cheilocystidia, few, narrowly lageniform to fusiform pleurocystidia, and clamp connections at the lower part of the stipe. *Hymenopellisutriformis* sp. nov. has mostly utriform pleurocystidia and 2-spored basidia. In the inferred phylogenies, the new species from this study formed distinct clades well supported by bootstrap proportions and posterior probabilities. The studied specimen affine to *H.orientalis* produced 2-spored basidia whereas published descriptions of other specimens mention 4-spored basidia. Moreover, the genetic distance between ITS sequences of this specimen and that of a *Hymenopellisorientalis* specimen from GenBank was 1.30–2.57%. Therefore, the conspecificity of our specimen with *H.orientalis* is uncertain, and additional specimens are needed to fully confirm its identity.

## ﻿Introduction

*Hymenopellis* R.H. Petersen, one of the genera in the Physalacriaceae Corner, was circumscribed by Petersen and Hughes (2010) as a new genus, covering those species with moist to glutinous pileus. It is the largest genus in the oudemansielloid/xeruloid complex and has a worldwide distribution. *Hymenopellis* species were previously classified in the section Radicatae of *Oudemansiella* (Clémençon 1979; Pegler and Young 1986; Yang et al. 2009). The presence of a pseudorrhiza separated O.sect.Radicatae from sect. Hygrophoroides (Clémençon 1979). The type species, *H.radicata* was first described by a British botanist, Richard Relhan, in 1780, under the name *Agaricusradicatus*, which is also synonymous with *Oudemansiellapseudoradicata* M.M. Moser, *Oudemansiellaradicata* (Relhan: Fr.) Singer and *Xerularadicata* (Relhan: Fr.) Dörfelt. There are around 50 species of *Hymenopellis* (He et al. 2019) of which 13 were first described from Asia (Petersen and Hughes 2010).

*Hymenopellis* is widely distributed in tropical and temperate regions (He et al. 2019). The majority of the literature on this genus has focused on Europe and the United States, where its taxonomy and distribution have been extensively researched. The most thorough study on *Hymenopellis* was done by Petersen and Hughes (2010), in which descriptions of all known species were provided. Out of the 50 described species, only 19 have sequences available in GenBank. The majority of sequences found in GenBank are from specimens collected in the eastern United States. Asian taxa are least represented (Petersen and Hughes 2010) with limited studies in this genus from Asian countries. Thirteen species of *Hymenopellis* have been recorded from Asia, of which 12 were first described from Asian countries. Six species were first described from temperate regions in China and Japan while another six species were described from tropical countries, namely *H.altissima* (Massee) R.H. Petersen from Singapore (as *Collybiaaltissima*), *H.bispora* (Natarajan & Purush.) R.H. Petersen, *H.keralae* R.H. Petersen & Manim. and *H.raphanipes* (Berk.) R.H. Petersen from India, *H.endochorda* (Berk. and Broome) R.H. Petersen from Sri Lanka (Petersen and Hughes 2010) and *H.neuroderma* (Pat.) R.H. Petersen from Vietnam (Petersen and Hughes 2010). In Thailand, only two species have been recorded, namely *H.raphanipes* (Petersen and Nagasawa 2006; Yang et al. 2009), and *H.radicata* (as *X.radicata*) (Chandrasrikul et al. 2011). However, the *H.radicata* recorded in Thailand has no associated sequence available. Thailand has a forested area of around 16.3 million ha (FAO 2020), a thriving habitat for diverse macrofungal species (Hyde et al. 2018). Many macrofungal species have been discovered in this country and many more remain to be introduced to science. Additional collections and further studies are necessary to improve our knowledge of Asian *Hymenopellis* taxonomy.

In this study, two new tropical species of *Hymenopellis* are introduced and a *Hymenopellis* specimen affine to *H.orientalis* is described from Thailand, adding to the limited number of Asian taxa.

## ﻿Materials and methods

### ﻿Sample collection and morphological observations

The specimens were collected from Chiang Rai and Chiang Mai provinces, Thailand during rainy season in June and August 2019. Photographs of the fresh samples were taken on the field, and information about habitat, habit, and other important features (e.g., color of the basidiomata, gills and stipe) of the specimen were noted. The basidiomata were carefully collected and kept in aluminum foil, labeled, and brought to the laboratory. Once in the laboratory, each specimen was photographed, measured, and described. Spore prints were collected on both black and white paper. Specimens were dried using a hot air dryer set to 45–50 °C for 24 hours. They were carefully labelled and stored in zip-lock bags to be used for further analyses. All samples were deposited in the Mae Fah Luang University fungarium (MFLU).

Macromorphological characters of the specimens (i.e., pileus, lamellae, and stipe) were described based on the fresh basidiomata. Naming of original colors was based on Methuen Handbook of Color, 3^rd^ ed. (Kornerup and Wanscher 1978). Preparation of macrofungal samples to describe micromorphological characters was based on the laboratory techniques by Clémençon (2009). Important features were examined using Motic SMZ-171 dissecting microscope and specific features were noted based on the terminology of Vellinga and Noordeloos (2001). Microscopic characters were observed using Nikon Eclipse Nἰ, DS-Ri2 compound microscope with dried samples rehydrated and mounted in water or in 3–5% KOH to retain original color. The prepared slides were stained with ammoniacal Congo Red to bring out hyaline structures. Specific features, i.e., basidiospores, basidia, cystidia and pellis, were drawn by free hand using standard microscopic techniques and described following the glossary of Vellinga and Noordeloos (2001). Dimension of at least 30 basidiospores per collection were measured in side view. The notation [A, B, C] preceding measurements of basidiospores, basidia and cystidia indicates the number (A) of those cells measured from the number (B) of basidiomata in the number (C) of collections. Measurements are presented as (a)b–c–d(e), where ‘a’ and ‘e’ are the extreme values, ‘b–d’ are the 5^th^ and 95^th^ percentiles, and ‘c’ is the average. Q represents the length/width ratio and Q*, the average value.

### ﻿DNA extraction, PCR and sequencing

DNA was isolated from samples taken from the dried specimens, using the Biospin Fungus Genomic DNA Extraction Kit (Bioer Technology, Hangzhou, China), following the manual’s procedure. The DNA loci amplified by PCR were the ITS region (including ITS1, 5.8S, ITS2) with the primers ITS1-F and ITS4 (White et al. 1990; Gardes and Bruns 1993), and nrLSU, with the primers LR0R and LR5 (Vilgalys and Hester 1990; White et al. 1990). PCR products were purified and sequenced in both directions, using the PCR primers, by Sangon Biological Engineering Technology and Services (Shanghai, China). The quality of each generated sequence read was checked using Bioedit Sequence Alignment Editor version 7.0.9.0 (Hall 1999) and sequence reads were assembled using SEQMan Pro software (DNA Star, Madison, USA).

### ﻿Phylogenetic analyses

Ten new sequences were generated in this study and were deposited in GenBank (Table 1). Each sequence was compared with sequences in GenBank (National Center for Biotechnology Information, NCBI) with the Basic Local Alignment Search Tool (BLAST). Forty-nine related accessions retrieved from GenBank, including three outgroup taxa, *Paraxerulaamericana* (Dörfelt) R.H. Petersen, *Strobilurusconigenoides* (Ellis) Singer and *X.pudens* (Pers.) Singer, were used to infer phylogenetic relationships with the newly generated sequences (Table 1). Outgroup taxa were chosen based on the ITS+nrLSU phylogeny in Hao et al. (2016). ITS and nrLSU were the only gene regions used to infer phylogenetic relationships with the newly generated sequences in this study. Other gene regions, especially the protein-coding ones, are very poorly represented in GenBank or non-existent, thus impossible to use for the analysis. The sequences were aligned using MAFFT version 7.450 (Katoh et al. 2019) on the server accessed at http://mafft.cbrc.jp/alignment/server/. TrimAl (Capella-Gutierrez et al. 2009) was used to eliminate ambiguously aligned positions from the alignments, using the strict mode option. The ITS and nrLSU alignments were 655 and 832 bp long, respectively. Phylogenetic tree inference was performed with partitioned maximum likelihood (ML) and Bayesian interference (BI) analyses. The two-character sets were ITS1+ITS2, and 5.8S+LSU. The best-fit nucleotide substitution model for ITS and nrLSU was selected with jModeltest version 2.1.10 (Darriba et al. 2012) based on the corrected Akaike Information Criterion (AICc). For the two gene regions, the HKY+G model was selected as the best model. ML analysis was performed through RAxML-HPC2 version 8.2.10 (Stamatakis 2014) on the web server CIPRES Science Gateway V. 3.3 (Miller et al. 2010) with GTRGAMMA as the model of evolution. The branch support was estimated with 1,000 rapid bootstrap replicates. The final alignment has been submitted to TreeBASE (submission ID 29774). For BI analysis, Markov Chain Monte Carlo (MCMC) sampling was performed using MrBayes v. 3.0b4 (Huelsenbeck and Ronquist 2001). Two runs of five simultaneous MCMC chains were run for 5,000,000 generations with trees and parameters sampled every 1,000^th^ generation, for a total of 10,000 samples. The first 25% of samples were discarded as burn-in phase. The remaining samples were used to calculate the majority rule consensus tree and associated posterior probabilities (PP). The trees were viewed using FigTree v1.4.2 (Rambaut 2012).

**Table 1.** List of sequences used in the phylogenetic analysis from GenBank with geographic origin and accession numbers of gene regions. The sequences newly generated for this study are in bold.

Species	Voucher/strain	Geographic	GenBank Accession No.		References
		origin	ITS	nrLSU	
*Hymenopelliscolensoi*	ZT12902	New Zealand	HM005139	HM005119	Petersen and Hughes (2010)
*H.colensoi*	PDD80639	China	–	AY960989	Unpublished
*H.furfuracea*	HKAS 93109	China	KX688223	KX688250	Hao et al. (2016)
*H.furfuracea*	TENN 61671	USA	GQ913362	HM005101	Petersen and Hughes (2010)
*H.furfuracea*	AFTOL-ID 538	USA	DQ494703	AY691890	Matheny et al. (2006); unpublished
*H.furfuracea*	TM03_474	Canada	–	EU522838	Porter et al. (2008)
*H.furfuracea*	JM98/155	China	AF321484	–	Mueller et al. (2001)
*H.furfuracea*	TENN 59876	USA	GQ913367	HM005126	Petersen and Hughes (2010)
*H.gigaspora*	NY REH 8676	Australia	GQ913357	HM005121	Petersen and Hughes (2010)
*H.gigaspora*	NY REH 8671	Australia	GQ913355	–	Petersen and Hughes (2010)
*H.gigaspora*	TENN 50056	Australia	GQ913358	–	Petersen and Hughes (2010)
*H.gigaspora*	TENN 50050	Australia	GQ913359	–	Petersen and Hughes (2010)
*H.hispanica*	05110401(SEST)	Spain	–	HM005082	Petersen and Hughes (2010)
*H.incognita*	TENN 58768	USA	GQ913424	HM005105	Petersen and Hughes (2010)
*H.incognita*	TENN 60228	USA	GQ913419	HM005104	Petersen and Hughes (2010)
*H.incognita*	EIU ASM10044	USA	GQ913422	–	Petersen and Hughes (2010)
*H.japonica*	HKAS 61674	China	KX688225	KX688252	Hao et al. (2016)
*H.japonica*	HKAS 83175	China	KX688226	KX688253	Hao et al. (2016)
*H.limonispora*	TENN 59438	USA	GQ913406	HM005133	Petersen and Hughes (2010)
*H.limonispora*	TENN 61379	USA	GQ913403	HM005134	Petersen and Hughes (2010)
*H.limonispora*	BIOUG24046-A02	Canada	KT695313	–	Telfer et al. (2015)
*H.megalospora*	DAOM196115			AF042649	unpublished
*H.orientalis*	HKAS 67938	China	KX688227	KX688254	Hao et al. (2016)
*H.orientalis*	HKAS 70323	China	KX688228	KX688255	Hao et al. (2016)
*H.orientalis*	TMI 2IX2002c1	Japan	GQ913396	–	Petersen and Hughes (2010)
*H.radicata*	TENN 62837	Sweden	GQ913375	HM005125	Petersen and Hughes (2010)
*H.radicata*	TENN 59329	Austria	GQ913380	–	Petersen and Hughes (2010)
*H.radicata*	TENN 60126	Russia	GQ913384	–	Petersen and Hughes (2010)
*H.radicata*	TENN 59223	France	GQ913392	–	Petersen and Hughes (2010)
H.radicatavar.bispora	TENN 57277	Sweden	GQ913379	HM005122	Petersen and Hughes (2010)
*H.raphanipes*	HKAS93070	China	KX688248	KX688275	Hao et al. (2016)
*H.raphanipes*	JBZ 2111002	China	KX688229	KX688256	Hao et al. (2016)
*H.raphanipes*	HKAS 93073	China	KX688231	KX688258	Hao et al. (2016)
*H.raphanipes*	HKAS 42555	China	GU980129	HM005108	Petersen and Hughes (2010)
*H.raphanipes* (2-spored)	TENN 59800	Thailand	GU980128	–	Petersen and Hughes (2010)
*H.raphanipes* (2-spored)	HKAS 42503	China	GU980130	–	Petersen and Hughes (2010)
*H.raphanipes* (as *O.chiangmaiae*)	TENN 59791	Thailand	KX964658	–	Hao et al. (2016)
*H.rubrobrunnescens*	TENN 52479	USA	GQ913371	–	Petersen and Hughes (2010)
*H.rubrobrunnescens*	TENN 52654	USA	GQ913372	HM005112	Petersen and Hughes (2010)
*H.rubrobrunnescens*	TENN 51262	USA	GQ913373	HM005113	Petersen and Hughes (2010)
*H.rugosoceps*	TENN 57307	USA	GQ913395	HM005116	Petersen and Hughes (2010)
*H.rugosoceps*	TENN 60604	USA	GQ913394	HM005117	Petersen and Hughes (2010)
*H.sinapicolor*	S.D. Russell MycoMap 6316	USA	MK560120	–	Unpublished
*H.sinapicolor* (holotype)	TENN 56566	USA	GQ913350	HM005118	Petersen and Hughes (2010)
***H.straminea***	**MFLU22-0138 holotype**	**Thailand**	**OP265162**	**OP265157**	**this study**
***H.straminea***	**MFLU22-0139**	**Thailand**	**OP265163**	**OP265158**	**this study**
*H.superbiens*	MEL2291946	Australia	GQ913360	HM005120	Petersen and Hughes (2010)
*H.trichofera*	MEL2293664	Australia	GQ913354	HM005129	Petersen and Hughes (2010)
***H.utriformis***	**MFLU22-0140 holotype**	**Thailand**	**OP265164**	**OP265159**	**this study**
***H.utriformis***	**MFLU22-0141**	**Thailand**	**OP265165**	**OP265160**	**this study**
*H.vinocontusa*	TMI 7669	Japan	GQ913370	–	Petersen and Hughes (2010)
**H.aff.orientalis**	**MFLU22-0142**	**Thailand**	**OP265166**	**OP265161**	**this study**
*Paraxerulaamericana*	CLO 4746	USA	HM005142	HM005094	Petersen and Hughes (2010)
*Strobilurusconigenoides*	TENN 61318	USA	GQ892821	HM005091	Petersen and Hughes (2010)
*Xerulapudens*	TENN 59208	Austria	HM005154	HM005097	Petersen and Hughes (2010)
“*Xerula* sp.”	BCC56836	Thailand	KX755407	KX755408	Sadorn et al. (2016)

Genetic distances between closely related sequences were measured from MAFFT aligned sequences. The genetic distances between ITS sequences were computed based on the combined ITS1 and ITS2 regions, excluding the 5.8S gene. For LSU, the full sequence between the primers LR0R and LR5 was used.

## ﻿Results and discussion

### ﻿DNA sequence analyses

The BLAST search results from the sequences of both loci (ITS and nrLSU) all matched with *Hymenopellis* taxa, thus indicating that all sequences generated from this study belong to this genus.

**Figure 1.**
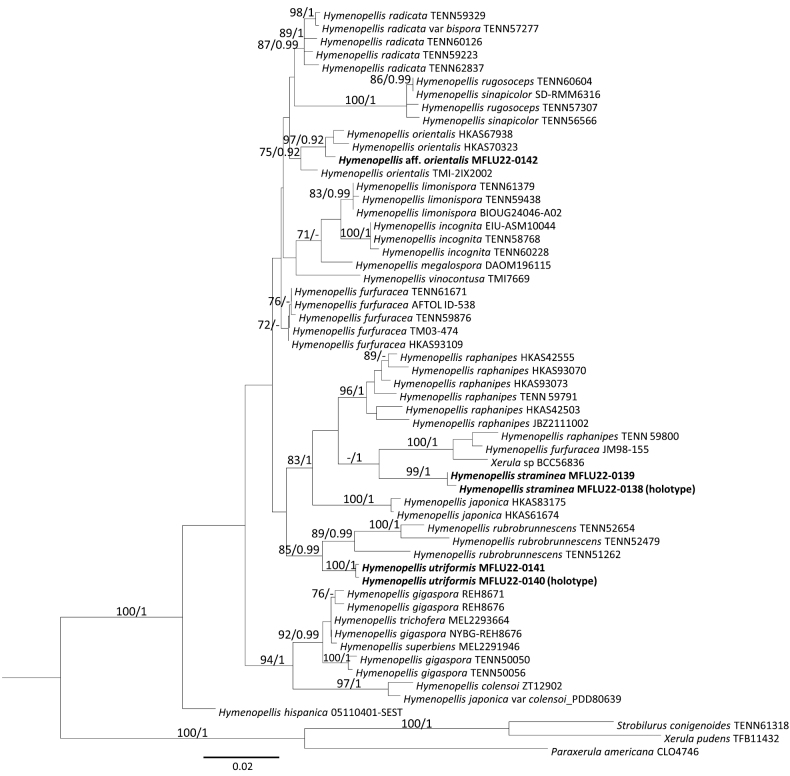
Phylogenetic tree generated from ML analysis of combined ITS and nrLSU data set for *Hymenopellis* with three outgroup species. Bootstrap support values (≥70%) and posterior probabilities (≥0.9) (BS/PP) are given above the branches. All termini are with species name and voucher ID, with the newly generated sequences from this study in bold.

In the combined ITS and nrLSU phylogeny, the new species *H.straminea*, represented by the specimens MFLU22-0138 (holotype) and MFLU22-0139, was monophyletic with 99% bootstrap support and 1.00 probability (Fig. 1). The ITS and nrLSU genetic distances between the two accessions were 0.52% (3/573) and 0.44% (4/905), respectively, and therefore are supported as conspecific. *Hymenopellisstraminea* was sister to the clade of *H.raphanipes* TENN 59800, *H.furfuracea* JM98-155 and “*Xerula* sp.” BCC56836 with 75% bootstrap support and a posterior probability of 1 (Fig. 1). The ITS genetic distances between *H.straminea* MFLU22-0138 (holotype) and the other accessions in the latter clade were 8.81% (49/556) for *H.raphanipes* TENN 59800, 8.83% (46/521) for *H.furfuracea* JM98-155 and 9.01% (51/566) for “*Xerula* sp.” BCC56836. The distance for nrLSU between *H.straminea* MFLU22-0138 and “*Xerula* sp.” BCC56836 was 2.73% (25/915).

“*Hymenopellisraphanipes*” TENN 59800 and “*H.furfuracea*” JM98-155 were separated from their respective species clades (Fig. 1). Therefore, it is likely that they were not identified correctly. The ITS genetic distance between *H.raphanipes* TENN 59800 and *H.raphanipes* TENN 59791 from Thailand was 8.68% (48/553). The ITS genetic distance between *H.furfuracea* JM98-155 and *H.furfuracea* HKAS 93109, both specimens from China, was 11.20% (57/509). The ITS genetic distances between specimens are much higher than the highest threshold value (3.0%) of species hypotheses in the Unite database (Nilsson et al. 2019), or the weighted average of the intraspecific ITS variability of Basidiomycota is 3.33% (Nilsson et al. 2008). The distances we observed therefore support separate species. BCC56836, on the other hand, was misidentified as “*Xerula* sp.” since it is clearly closely related to *Hymenopellis* species. However, BCC56836 is a culture collection only published for its bioactivity, without a corresponding herbarium specimen. Therefore, its morphology cannot be checked. The ITS genetic distances between “*Xerula* sp.” BCC56836 and *H.raphanipes* TENN 59800 and *H.furfuracea* JM98-155 were 3.91% (22/562) and 3.8% (20/527), respectively, while the ITS genetic distance between *H.raphanipes* TENN 59800 and *H.furfuracea* JM98-155 was 2.67% (14/527). It is possible that *H.raphanipes* TENN 59800, *H.furfuracea* JM98-155 and “*Xerula* sp.” BCC56836 are conspecific but further taxonomic studies, especially morphological comparisons among specimens belonging to this clade, are needed to confirm this assumption. Also, a detailed study of the holotypes of *H.raphanipes* and *H.furfuracea* is needed to confirm which of the sequenced specimens identified as those two species, if any, actually belong to them.

*Hymenopellisutriformis* sequences MFLU22-0140 (holotype), MFLU22-0141 separated from the clade of *Hymenopellisrubrobrunnescens* with 77% bootstrap support and 0.98 probability. The ITS genetic distance between the holotype *H.utriformis* MFLU22-0140 and *H.rubrobrunnescens* TENN 51262 is 8.06% (46/571), thus are considered to be separate species. The two sequences of *H.utriformis* (MFLU22-0140, MFLU22-0141) generated from this study joined together and are well-supported with 100% bootstrap support and 1.00 probability. The ITS and nrLSU genetic distances between the two generated sequences are 0.69% (4/577) and 0.22% (2/890), respectively, thus considered as conspecific.

The *Hymenopellis* sp. MFLU22-0142 fell into the clade of *H.orientalis* with 64% bootstrap support and 0.92 posterior probability. The ITS genetic distances between *Hymenopellis* sp. MFLU22-0142 from this study and *H.orientalis* TMI-2IX2002c1 and HKAS70323 are 2.57% (14/545) and 1.30% (7/539), respectively.

ITS1 and ITS2 are fast-evolving loci and are very useful in species delimitation in *Hymenopellis*. The often advocated 3% threshold to separate interspecific and intraspecific ITS genetic distances worked well for the two new species here described, with interspecific distances from their closest relatives being well above this value. However, for the specimen related to *Hymenopellisorientalis*, while the ITS genetic distance between our specimen and the Japanese specimen was lower than 3%, morphological differences were observed. More specimens related to *H.orientalis* must be studied to determine if they belong to one or more than one species. The 3% threshold should not be considered as universal. Some Basidiomycota genera have indeed been reported to exhibit lower intraspecific ITS variability such as *Amanitamuscaria* (0.9%) and *Boletusedulis* (0.3%) (Nilsson et al. 2008).

### ﻿Taxonomy

#### ﻿ Hymenopellis straminea

Taxon classificationFungiAgaricalesPhysalacriaceae

Niego & Raspé sp. nov.

1EA1F6AE-2E8F-5375-BDEC-957A388A0964

MycoBank No: 845750

Facesoffungi Number: FoF12896

Figs 2 , 3

##### Type.

Thailand. Chiang Rai Province: Mae Fah Luang District, elevation 1,110 m, tropical hill forest with grass dominated by *Castanopsis* and *Lithocarpus* trees, 14 June 2019, A.G. Niego, MFLU22-0138 (holotype); GenBank OP265162-ITS, OP265157-nrLSU.

##### Etymology.

The name refers to the straw-yellow color of the pileus.

##### Diagnosis.

Differentiated from similar *Hymenopellis* species by the small (< 5 cm), straw-yellow pileus and lamellae without decurrent tooth.

##### Description.

***Basidiomata*** small-sized. ***Pileus*** 35–45 mm diam., circular in polar view, in side view convex to applanate, straw-yellow or buff (4B5) evenly colored but darker when young; surface dry to viscid, sticky when wet, non-hygrophanous, rugulose, moderately wrinkled; margin decurved to plane, translucent striate; context white, unchanging when cut, consistency rubber-like. ***Lamellae*** 4–5 mm broad, thick, white, ventricose, adnate with no distinct decurrent tooth, spacing > 1 mm; lamellar margin even; lamellulae present, regularly arranged, in 2 (3) tiers. ***Stipe*** 65–85 × 3–4 mm, central, cylindrical, mostly equal, thickened at the base, light brown, lighter (5A2) from the pileus becomes yellowish brown (5D5) towards the base, surface dry, appressed squamulose especially towards the base, fistulose; context white, unchanging when cut; pseudorrhiza present. ***Spore print*** white. ***Smell*** indistinct. ***Taste*** mild.

**Figure 2.**
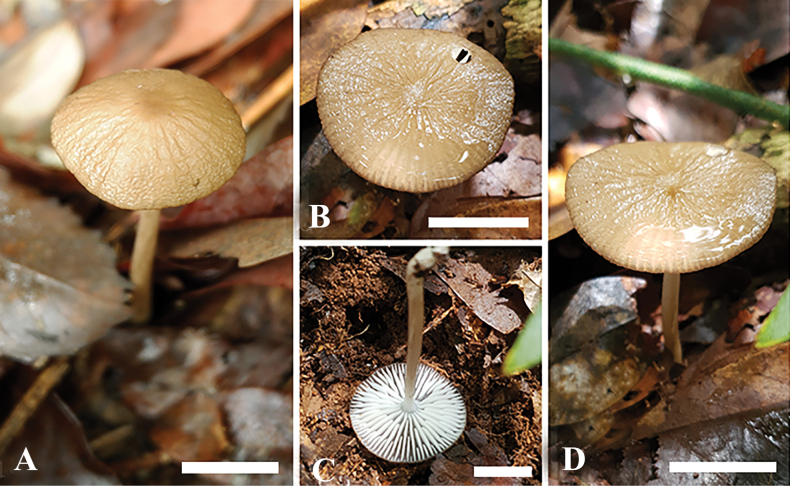
Basidiomata of *Hymenopellisstraminea* MFLU22-0138, holotype **A, B, D** top view of basidiomata **C** view of lamellae. Scale bar: 3 cm (**A–D**) Photographs by A.G. Niego.

***Basidiospores*** [60,2,2] (9)10.2–12.8–14.5(15) × (8)8.5–11–11.5(12) µm (Q = 1.0–1.3, Q* = 1.2), subglobose to ellipsoid, thin-walled, hyaline in 5% KOH. ***Basidia*** [30,2,2] (35)36–42.8–57(60) × 12–14.3–20 µm (Q = 2.7–3.3, Q* = 3.0), tetrasporic, clavate, without clamp connection; contents grossly granular. ***Cheilocystidia*** [30,2,2] (21)26–47–73.5(74) × (6)9.5–12.5–18(21) µm (Q = 2.1–6.2, Q* = 3.8), numerous, grouped together, pedunculate, narrowly lageniform, clavate to broadly clavate, fusiform, smooth, thin-walled, hyaline in 5% KOH. ***Pleurocystidia*** [30,2,2] (48.5) 55–87–136 (168) × (15.5) 16–21.5–29 (32.5) µm (Q = 2.5–5.8, Q* = 3.6), mostly narrowly lageniform but can also be fusiform, smooth, thin-walled, hyaline in 5% KOH. ***Hymenophoral trama*** irregular, made of thin-walled, hyaline hyphae. ***Pileipellis*** an epithelioid hymeniderm with some extended pileal hairs; terminal elements (24.5)25.5–31–36(43) × (12)12.5–14–17(18) µm, with scattered intracellular light brown (6D8) pigment in 5% KOH. ***Stipitipellis*** a cutis; hyphae (7.5)8–9.5–11.5(12) µm wide, with intracellular light brown (6D8) pigment in 5% KOH. ***Clamp connections*** were seen in the lower part of the stipe.

**Figure 3.**
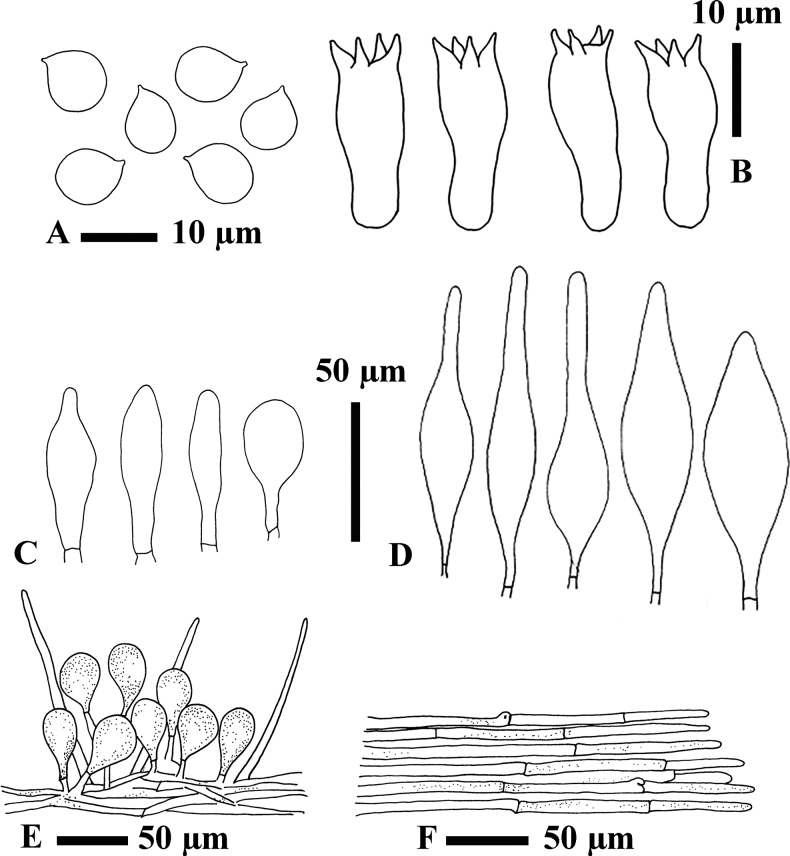
Micromorphological features of *H.straminea* MFLU22-0138, holotype **A** basidiospores **B** basidia **C** cheilocystidia **D** pleurocystidia **E** pileipellis **F** stipitipellis.

##### Habitat and distribution.

Solitary, in tropical hill forest of Chiang Rai Province, Thailand.

##### Additional specimen examined.

Thailand. Chiang Rai Province: Mae Fah Luang District, elev. 1,100 m, tropical hill forest, 14 June 2019, A.G. Niego, MFLU22-0139; GenBank OP265163-ITS, OP265158-nrLSU.

##### Notes.

*Hymenopellisstraminea* is quite similar to *H.megalospora* (Clem.) R.H. Petersen, the latter having usually small pileus (<50 mm) but *H.megalospora* can sometimes reach up to 120 mm diam. The color of *H.megalospora* may range from disc deep olive brown to “buckthorn brown” (5D6) to pale ochraceous buff (4A2), to nearly white, with or without a darker center. The stipe of *H.megalospora*, however, is quite longer (70–250 × 2–3 mm), and the lamellae are strongly decurrent, which is not evident in *H.straminea*. Moreover, *H.megalospora* has larger basidiospores (15–21 × 8–12 µm) which are finely dimpled or pitted (Petersen and Hughes 2010).

*Hymenopellisstraminea* is also quite similar to some specimens of *H.furfuracea* (Peck) R.H. Petersen in having a broadly convex to nearly flat pileus with bald and moderately wrinkled surface. *Hymenopellisfurfuracea* basidiomata are more diverse in color (dark brown to gray brown or yellow brown) and size (very small to large). Lamellae also have slight decurrent tooth (Yang et al. 2009; Petersen and Hughes 2010). *Hymenopellisstraminea* on the other hand is consistently small in pileus size (35–45 mm), evenly straw-yellow.

Finally, *Hymenopellisraphanipes* is different from the new species by having mostly dark colored basidiomata but they can sometimes be “buckthorn brown” (5D6), and also vary in size from small to large (Petersen and Hughes 2010). Strains of *H.raphanipes* also have 2- and 4- spored basidia. *H.straminea* basidia, however, are always 4-spored. When compared with *H.raphanipes* TENN 59800, the herbarium specimen with which *H.straminea* formed a clade, the morphology is quite different. The most obvious difference is the much bigger basidiospores of *H.raphanipes* TENN 59800 [(13.7) 14–15.8–17 (18) × (11) 12.5–13.3–14 (15) µm]. The terminal elements of the pileipellis of *H.raphanipes* TENN 59800 are also larger [(20)23–37–50(70) × (10)11–14.7–17.5(21) µm]. Those morphological differences, together with the high genetic distance in the clade, support that *H.straminea* is a novel species.

#### ﻿ Hymenopellis utriformis

Taxon classificationFungiAgaricalesPhysalacriaceae

Niego & Raspé sp. nov.

80F7E5D0-89CC-51E2-85C5-E1ED3B7955D2

MycoBank No: 845751

Facesoffungi Number: FoF12895

Figs 4 , 5

##### Type.

Thailand. Chiang Mai Province: Mae Taeng District, elev. 400 m, tropical deciduous forest, 09 August 2019, A.G. Niego, MFLU22-0140 (holotype); GenBank OP265164-ITS, OP265159-nrLSU.

##### Etymology.

The name refers to the most common utriform or narrowly utriform pleurocystidia of the type specimen.

##### Diagnosis.

Differentiated from other *Hymenopellis* species by the moist to viscid, light brown pileus, mostly utriform pleurocystidia and 2-spored basidia.

##### Description.

***Basidiomata*** small-sized to large. ***Pileus*** 25–95 mm diam., circular in polar view, in side view broadly convex to plane to slightly depressed, light brown (5C5), moist to viscid, non-hygrophanous, rugose surface, radially wrinkled with age; margin plane to decurved, translucent striate; context cream (1A3) to white, unchanging when cut, consistency rubber-like. ***Lamellae*** 4–8mm broad, adnexed, ventricose, white to cream (1A3), spacing > 1 mm; lamellar margin even; lamellulae present, in 2 tiers. ***Stipe*** 50–185 mm × 4–12 mm, central, cylindrical, mostly equal, thickened at the base, off-white to light brown (5A2) from the pileus becomes darker (5D4) towards the base, surface dry, appressed squamulose especially towards the base, narrowly fistulose; context white, unchanging when cut; pseudorrhiza present. ***Annulus*** and ***volva*** absent. ***Spore print*** white. ***Smell*** indistinct. ***Taste*** slightly sweet.

**Figure 4.**
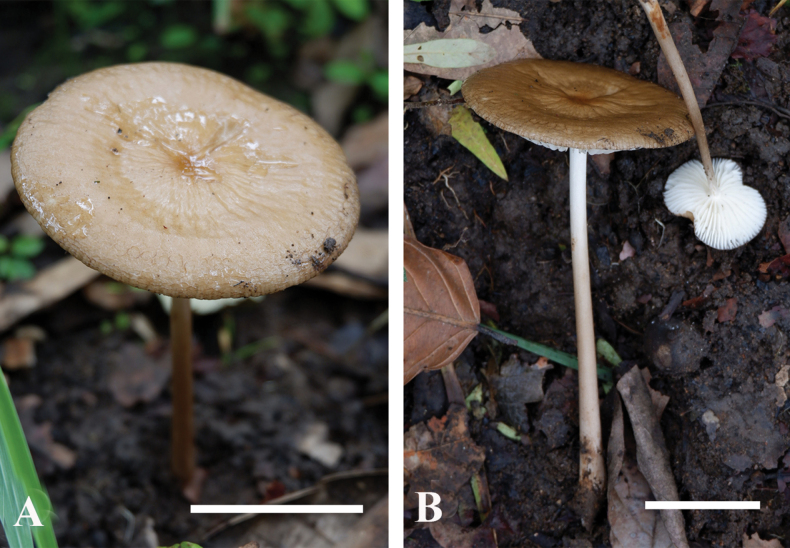
**A, B** basidiomata of *Hymenopellisutriformis* MFLU22-0140, holotype. Scale bars: Photographs by A.G. Niego.

***Basidiospores*** [60,3,1] (11.7) 12–13.7– 16.7 (17) × (9.3) 10.1–11.4–12.6 (12.7) µm (Q = 1.0–1.5, Q* = 1.2), subglobose to ellipsoid, thin-walled, hyaline in 5% KOH. Basidia [30,3,1] (36) 36.7–38.1–39.2 (39.5) × (9.4) 11.3–11.6–12.8 (13) µm (Q = 3.0–4.0, Q* = 3.3), 2–spored, clavate, without clamp connection. ***Cheilocystidia*** [30,3,1] (31) 38–52–64 (67.7) × (8.6) 9–13.2–18 (18.5) µm (Q = 3.1–5.0, Q* = 3.9), numerous, grouped together, pedunculate, narrowly clavate to clavate, conical, narrowly utriform to utriform, smooth, thin-walled, hyaline in 5% KOH. ***Pleurocystidia*** [30,3,1] (83) 88–116.3–131 (174) × (22) 22.5–30–35 (37.5) µm (Q = 2.9–5.4, Q* = 3.9) scattered, narrowly utriform to utriform, smooth, thin-walled, hyaline in 5% KOH. Hymenophoral trama irregular, made of thin-walled, hyaline hyphae. ***Pileipellis*** an epithelioid hymeniderm; terminal elements 28–52–76 × 11–13.7–17.5 µm with few scattered intracellular light brown (6D8) pigment in 5% KOH. ***Stipitipellis*** a trichoderm, terminal elements 28–52–76 × 11–13.7–17.5 µm, with intracellular light brown (6D8) pigment in 5% KOH. Clamp connections not seen.

**Figure 5.**
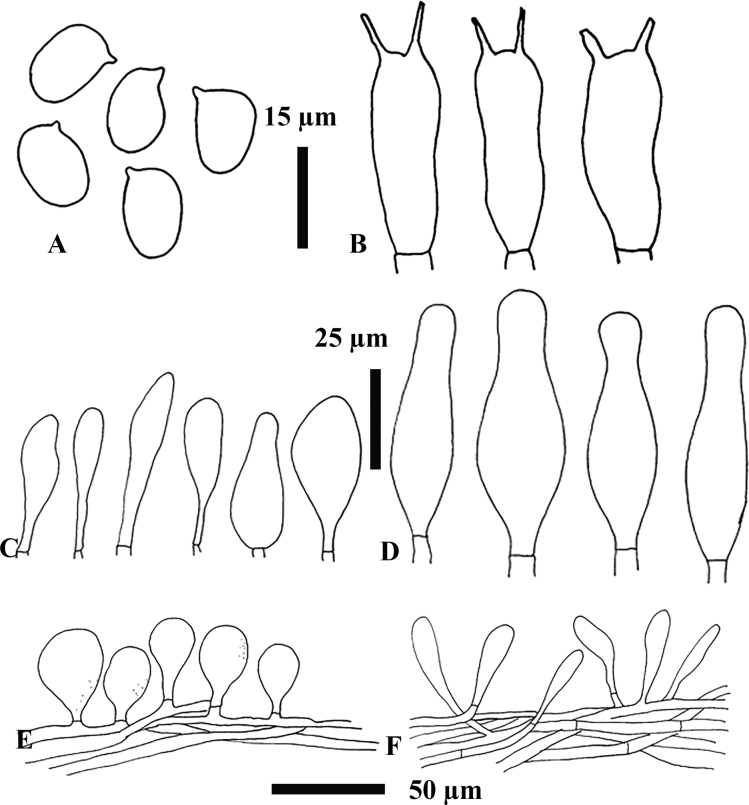
Micromorphological features of *H.utriformis* MFLU22-0140, holotype **A** basidiospores **B** basidia **C** cheilocystidia **D** pleurocystidia **E** pileipellis **F** caulocystidia.

##### Habitat and distribution.

Solitary to clustered, in soil covered with degrading leaves and other organic matters, in deciduous forest of Chiang Mai Province, Thailand.

##### Additional specimen examined.

Thailand. Chiang Mai Province: Mae Taeng District, elev. 375 m, tropical deciduous forest, 09 August 2019, A.G. Niego, MFLU22-0141; GenBank OP265165-ITS, OP265160-nrLSU.

##### Notes.

*Hymenopellisutriformis* is similar to *H.rubrobrunnescens* (Redhead, Ginns & Shoemaker) R.H. Petersen, having small to large but gracile basidiomata. The color is “tawny olive” (5C5) with rugose to rugulose surface.

*Hymenopellisradicata* (Relhan) R.H. Petersen, as described by Petersen and Hughes (2010), is similar to *H.utriformis* in having large basidiomata and a mid-brown (5–6D, 5–6E5–8, 5E7, 4E7, 6D3) pileus which is radially wrinkled. Both species are rather moist to viscid. However, *H.radicata* stipe is longitudinally lined, usually twisted while its cheilocystidia are clavate to subcapitate when young, broadly cylindrical, jar-shaped to occasionally mammillate when mature. The cheilocystidia of *H.utriformis* were more diverse in shapes. Pleurocystidia of *H.radicata* are strongly inflated, bluntly rounded to hemispherical apically, narrowly utriform to utriform whereas *H.utriformis* have narrowly utriform to utriform pleurocystidia only.

Other species similar to *H.utriformis* found in Asia are *H.furfuracea* and *H.raphanipes*, both having medium to large basidiomata but with more diverse pileal colors (Petersen and Hughes 2010). *Hymenopellisfurfuracea* basidia are tetrasporic while those of *Hymenopellisraphanipes* can be 2-spored, except for the synonymized *H.chiangmaiae*, which is the tetrasporic form from Asia. *Hymenopellisutriformis* basidia, however, are strictly 2-spored.

#### ﻿ Hymenopellis aff. orientalis

Taxon classificationFungiAgaricalesPhysalacriaceae

(R.H. Petersen & Nagas.) R.H. Petersen

BA41D4FE-C29C-5886-AECB-FE6A743B234B

MycoBank No: 800798

Figs 6 , 7

##### Description.

***Basidiomata*** small-sized. ***Pileus*** 15 mm diam., convex with an umbo, light brown (4B4) with slightly darker color at the center brown (5C5), paler toward margin, non-hygrophanous, slightly viscid, appressed-squamulose surface, radially wrinkled; margin inflexed, translucently striate; context white to cream (1A3), unchanging when cut, consistency rubbery. ***Lamellae*** subventricose, rubbery to rather soft, 3 mm broad, 0.5 mm thick, adnate with slight decurrent tooth, white, subdistant (1 mm apart); lamellar margin finely fimbriate; lamellulae present, in 1–2 tiers. ***Stipe*** 67 mm × 2.5 mm, central, cylindrical, tapered upwards, slightly clavate base, whitish to light brown (4A2) from the pileus to slightly darker (4B4) downwards, stuffed to fistulose; surface dry, fibrillose, finely dotted to minutely appressed-squamulose especially towards the base, which is covered with white tomentum; context white, unchanging when cut; pseudorrhiza present, 60 mm long. ***Annulus*** and ***volva*** absent. ***Spore print*** white. ***Smell*** indistinct. ***Taste*** mild.

**Figure 6.**
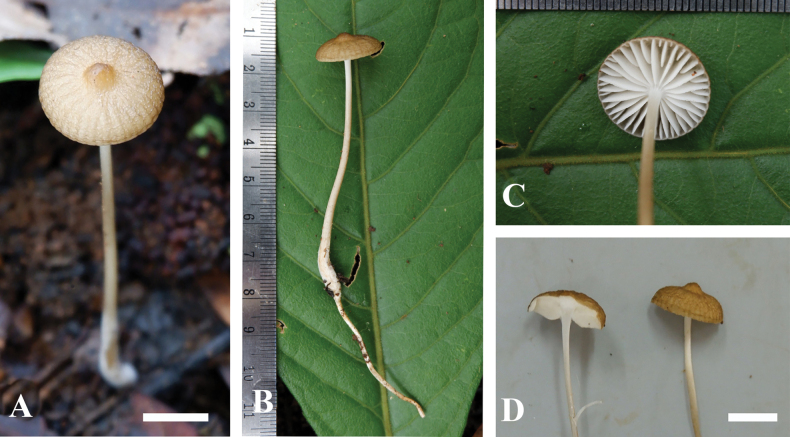
Basidioma of Hymenopellisaff.orientalis MFLU22-0142 **A** top view **B** side view **C** view of lamellae **D** side view of context. Scale bar: 1.0 cm (**A, D**). Photographs by A.G. Niego.

***Basidiospores*** [50,1,1] (13.5) 15–16.4–17.5 (18) × (9.5)–13–13.5 (14.5) μm (Q = 1.2–1.4, Q* = 1.3), broadly ellipsoid to ellipsoid, ovoid, obovoid, thin-walled, delicately puckered, hyaline in 5% KOH. ***Basidia*** [30,1,1] (39) 41–48.3–59 (61) × (10)10.5–12–15 (16) μm (Q = 3.1–5.0, Q* = 4.0), bisporic, narrowly to broadly clavate, hyaline in 5% KOH. ***Cheilocystidia*** [15,1,1] (49) 53–78.2–110 (118) × (8.7) 12–16.6–23 (26) µm, (Q = 2.9–6.6, Q* = 4.8), numerous, short-pedicellate, conical, fusiform, narrowly clavate, narrowly cylindrical, narrowly lageniform to lageniform, often clamped, smooth, thin-walled, hyaline in 5% KOH. ***Pleurocystidia*** [30,3,1] (71) 75–106.7–130 (132) × (24) 24.5–28.5–36 (37.5) µm (Q = 2.8–5.4, Q* = 3.8), fusiform, clavate, narrowly clavate, utriform, narrowly utriform, rounded apex, smooth, firm-walled, hyaline in 5% KOH. ***Hymenophoral trama*** irregular, made of thin-walled, hyaline hyphae. ***Pileipellis*** a hymeniderm; terminal elements (26.5)27–34.2–42(46) × (12)13–17–22(25.5) µm, with scattered intracellular light brown (6D8) pigment in 5% KOH. ***Stipitipellis*** an intricate trichoderm; hyphae (3.3)3.5–5.0–6.0(6.2) µm wide, hyaline in 5% KOH. ***Clamp connections*** not observed.

**Figure 7.**
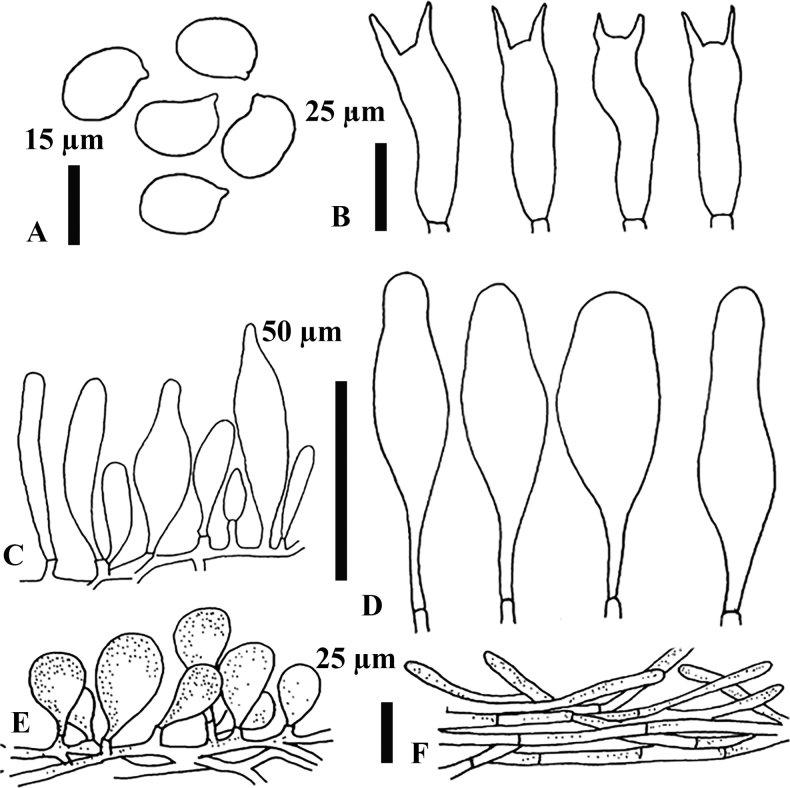
Micromorphological features of Hymenopellisaff.orientalis MFLU22-0142 **A** basidiospores **B** basidia **C** cheilocystidia **D** pleurocystidia **E** terminal elements of pileipellis **F** stipitipellis.

##### Habitat and distribution.

Solitary, on the soil covered with litter, in tropical hill forest of Chiang Mai Province, Thailand.

##### Specimen examined.

Thailand. Chiang Mai Province: Mae Taeng District, Ban Pa Daeng, elev. 1,110 m, tropical evergreen hill forest, 08 August, 2019, A.G. Niego, MFLU22-0142; GenBank OP265166-ITS, OP265161-nrLSU.

##### Notes.

The specimen described in this study is morphologically quite similar to *H.orientalis*, which was first described from Japan (Petersen and Nagasawa 2006). However, it has a smaller pileus (15 mm diam.). It also produces 2-spored basidia whereas those of the holotype are 4-spored. The ITS genetic distances from the two most closely related *H.orientalis* TMI-2IX2002c1 and HKAS70323 were 2.57% and 1.30%, respectively. Such distances may be compatible with conspecificity. However, some morphological differences were noted, but based only on the single specimen we collected. Therefore, we use the name H.aff.orientalis until additional collections are available from tropical and temperate Asia to ascertain its taxonomic identity and properly describe it if it is confirmed to be a new species different from *H.orientalis*.

## Supplementary Material

XML Treatment for Hymenopellis straminea

XML Treatment for Hymenopellis utriformis

XML Treatment for Hymenopellis aff. orientalis

## ﻿Additional information

### Author ORCIDs

Allen Grace T. Niego https://orcid.org/0000-0001-8125-5061

Naritsada Thongklang https://orcid.org/0000-0001-9337-5001

Kevin D. Hyde https://orcid.org/0000-0002-2191-0762

Olivier Raspé https://orcid.org/0000-0002-8426-2133

### Data availability

All of the data that support the findings of this study are available in the main text, or in publicly accessible data repositories, as indicated in the text.
